# Multi-valued and Fuzzy Logic Realization using TaOx Memristive Devices

**DOI:** 10.1038/s41598-017-18329-3

**Published:** 2018-01-08

**Authors:** Debjyoti Bhattacharjee, Wonjoo Kim, Anupam Chattopadhyay, Rainer Waser, Vikas Rana

**Affiliations:** 1 School of Computer Science and Engineering, Nanyang Technological University, Singapore, Singapore; 2 Peter Grünberg Institut 7, Forschungszentrum Jülich GmbH, 52425 Jülich, Germany; 3 School of Physical and Mathematical Sciences, Nanyang Technological University, Singapore, Singapore; 4 Institut für Werkstoffe der Elektrotechnik II, RWTH Aachen University, 52074 Aachen, Germany

## Abstract

Among emerging non-volatile storage technologies, redox-based resistive switching Random Access Memory (ReRAM) is a prominent one. The realization of Boolean logic functionalities using ReRAM adds an extra edge to this technology. Recently, 7-state ReRAM devices were used to realize ternary arithmetic circuits, which opens up the computing space beyond traditional binary values. In this manuscript, we report realization of multi-valued and fuzzy logic operators with a representative application using ReRAM devices. Multi-valued logic (MVL), such as Łukasiewicz logic generalizes Boolean logic by allowing more than two truth values. MVL also permits operations on fuzzy sets, where, in contrast to standard crisp logic, an element is permitted to have a degree of membership to a given set. Fuzzy operations generally model human reasoning better than Boolean logic operations, which is predominant in current computing technologies. When the available information for the modelling of a system is imprecise and incomplete, fuzzy logic provides an excellent framework for the system design. Practical applications of fuzzy logic include, industrial control systems, robotics, and in general, design of expert systems through knowledge-based reasoning. Our experimental results show, for the first time, that it is possible to model fuzzy logic natively using multi-state memristive devices.

## Introduction

Claude Shannon, in his landmark work^1^, demonstrated that the two-valued logic system developed by George Boole^2^, can be mimicked through operations of an electrical circuit. This resulted in widespread adoption of two-valued switching algebra or Boolean algebra. For every digital device in the modern world, Boolean algebra is used to perform the underlying computation. Boolean logic uses two truth values, *true* and *false*, even though in real life, we often require more than two truth values for describing an event. For example, we describe a day as ‘sunny’, ‘partly clouded’ or ‘clouded’, which means that the element weather cannot be discretely classified into the set ‘sunny’ or ‘clouded’ To capture such phenomena and their underlying logical process, *multi-valued* logic system was introduced.

In 300 BC, Aristotle proposed the *principle of non-contradiction* which ruled out simultaneous existence of two contradictory propositions^3^. This principle, also known as the *law of excluded middle*, is one of the classical laws of thought. In modern times, Jan Łukasiewicz introduced a third truth value, thereby first formally studying the field of multi-valued logic (MVL) in 1920^4^. In the following year, Emil Post published a system of functionally complete MVL algebra with additional truth degrees (*n* ≥ 2), ‘chained Post algebra’^5^. Later on, finite-valued Łukasiewicz logic was rigorously axiomatized and extended to arbitrarily many truth values^6,7^. Applications of MVL can be found in linguistics^8^, circuit simulation and manufacturing testing of digital circuits^9^.

An important offshoot of MVL that permits *inference under vagueness* and allows real-valued member elements is Fuzzy logic. The term *fuzzy logic* was introduced by Lotfi A. Zadeh in context of fuzzy set theory^10^. In contrast to the classical logic systems that adheres to a set of elements with *crisp* truth values, fuzzy logic operates on fuzzy sets. In a fuzzy set, elements of the set can have a degree of membership. Operators from a MVL, e.g., Łukasiewicz logic, can be applied to the fuzzy set, akin to how Boolean logic operators are applied to the crisp set. In fuzzy logic, a linguistic model is built from a set of IF–THEN rules which describe the control model. Mamdani Warren demonstrated that fuzzy logic could be used for developing operational automated control systems^11^ and clinical practice decision support systems^12^. The most well known application of fuzzy logic based control system was deployed in Sendai Subway 1000 series subway trains in Japan for speed control^13^. The fuzzy controller based train had a higher relative smoothness of the starts and stops when compared to other trains, and was 10% more energy efficient than human-controlled accelerated trains. Further applications of fuzzy logic include expert systems^14^, robotics^15^ and diverse sub-domains of machine intelligence^16^.

Despite these wide ranging applications of MVL, the present computing technology is heavily based on Boolean logic. There have been prior studies on porting MVL to digital and analog circuits with promising results. MVL has been demonstrated to improve energy-efficiency by reducing the switching activity of VLSI interconnects^17,18^. MVL based arithmetic circuits are simpler and more efficient over corresponding Boolean logic based implementations^19–21^. However, a limiting factor of MVL realization has been the inherent representation of information in binary format in semiconductor devices, thereby forcing a designer to switch between logic formats, which was clearly an inefficient solution. In this manuscript, we leverage the multi-state memristive devices which can inherently operate in the multi-valued domain. The multi-state memristive devices used in this experiment are Redox-based resisitve switches (ReRAMs), which are considered as one of the most promising emerging non-volatile memory technologies^22–24^. Implementation using passive crossbar configuration enables ultra-dense 4*F* ^2^ integration. Recently, *TaO*_*x*_ based ReRAMs draw significant attention due to excellent performance in term of high endurance (>10^12^)^25^, long retention (10 years)^26^, multi-level switching capability (3-bit)^27^ and fast read/write speed of below 200 *ps* ^28^. Besides the memory applications, ReRAM based passive crossbar arrays offer the implementation of memory-intensive computing paradigms, i.e. the logic operations are directly processed in the memory and arithmetic tasks. This merges the boundaries between memory and arithmetic logic units and eases the von-Neumann-bottleneck for computation^29^. Furthermore, memristive crossbar arrays can enable the multi-parallel search algorithms for pattern recognition tasks, widely required for neuromorphic applications^30^.

The current paper reports the first implementation of Łukasiewicz logic using the ReRAM-based memristive devices. We do not impose any theoretical limit on the number of states for the memristive devices^31^ and hence this work can be used for realizing any application that uses finite-valued Łukasiewicz logic family *L*_*n*_. Before going to the implementation details, we briefly review the basics of Łukasiewicz logic. Formally, a finitely-valued Łukasiewicz logic family *L*_*n*_ can be defined over the following truth values.

1 $${L}_{n}=\{\mathrm{0,}\,\frac{1}{n-1},\frac{2}{n-1},\,\ldots ,\,\frac{n-2}{n-1},\,1\}$$

The designated truth value is 1. Implication (IMP→) and negation (NEG¬) are the two operators used in Łukasiewicz logic, defined through the following functions.

2 $$\neg u=1-u$$

3 $$u\to v=min\mathrm{\{1,}\,1-u+v\}\,u,v\in {L}_{n}$$

In this paper, we also present a detailed case study to realize fuzzy logic control using Łukasiewicz logic and the implementation of a fuzzy logic controller using multi-state ReRAM crossbar arrays.

## Results

### Device Properties

**Figure 1:**
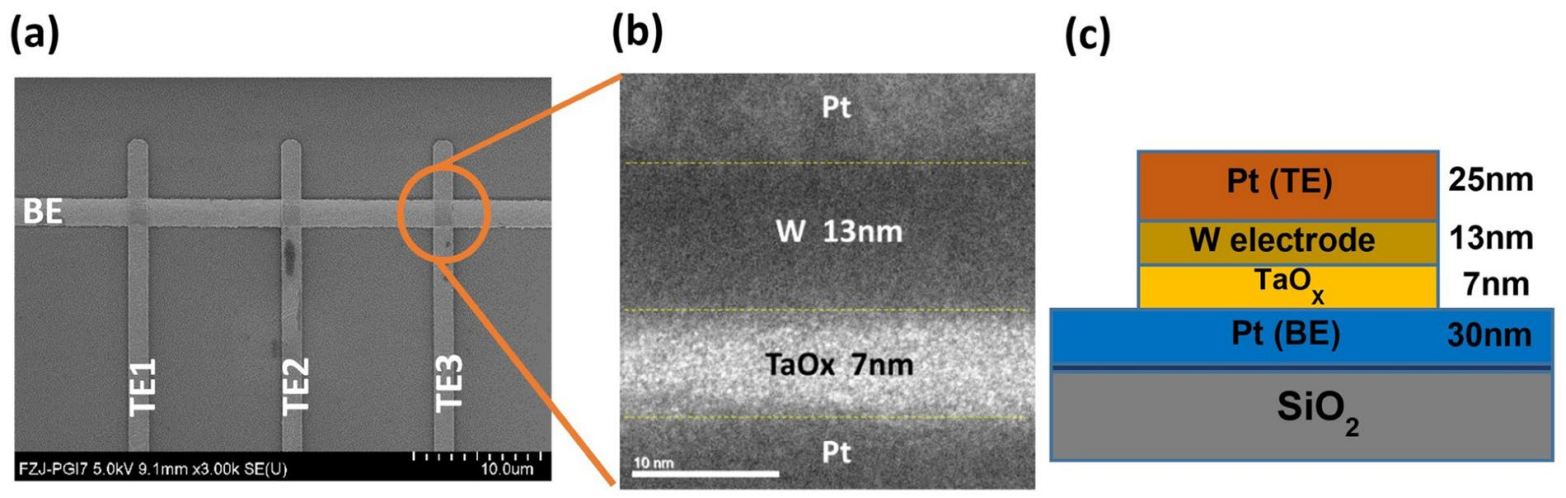
Resistive switching device structures. (**a**) Scanning electron microscopy image of 1 × 3 array, with 2 *μm* × 2 *μm* device. (**b**) Tunnelling electron microscopy image of a single device cross-section with 7 *nm*-thick *TaO*_*x*_ switching layer and 13*nm*-thick tungsten ohmic electrode. (**c**) Schematic diagram of the single resistive device.

**Figure 2:**
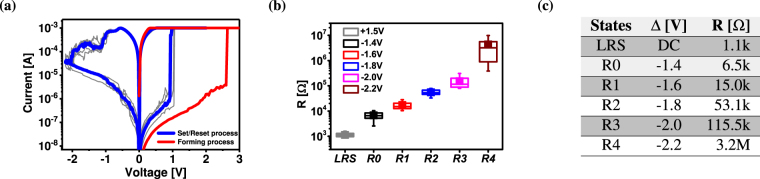
Device characteristics. (**a**) Current–Voltage (*I*–*V*) characteristics and electroforming curve of *TaO*_*x*_–based ReRAM device. (**b**) Resistance distribution based on median, obtained by pulse duration of 200 *ns* and amplitude in the range of −1.4*V* to −2.2*V* (0.2*V* steps) enable highly accurate resistive state control. (**c**) Mean value of final resistance is estimated based on measurements of 4 devices for 10 cycles per state.

In this work, 2 *μm* × 2 *μm* *Pt*/*W*/*TaO*_*x*_/*Pt* cross-point bipolar memristive devices arranged in word structure have been fabricated. Figure 1 shows the scanning electron microscope and transmission electron microscopy image of the devices used in this experiment. The ReRAM device stack of 25 *nm Pt*/13 *nm W*/7 *nm TaO*_*x*_/30 *nm Pt* is depicted in Fig. 1(b). Figure 1(c) shows the schematic of a single device along with the details of the stacked layers. Figure 2(a) shows the typical *I*–*V* characteristics of the ReRAM device with set current compliance of 1.0 *mA*, along with the electroforming curve. After the electroforming process, the device was toggled to high resistance state by applying the reset voltage. The maximum applied voltage |*V*_*stop*_| during RESET process defines the final resistive state of the device. This feature is also used in pulse mode operation, and can thus be used in memory and logic operations for controlling the multi-level states. To enable highly reproducible RESET operation, we have always applied a DC SET operation before each pulsed RESET operation (200 *ns*). Note that a nanosecond pulsed SET operations are also feasible, but have not been applied in this work. Figure 2(b) shows a very tight resistance distribution of low resistance state (LRS) and six multi-level resistive states. This confirms the excellent switching properties of these devices. For the multi-level programming, we have split the total applied amplitude across the bottom electrode and the top electrode. A fixed positive amplitude (+0.7*V*) is assigned at the bottom electrode while a varying negative amplitude (−0.7*V* to −1.5*V*) is applied to the top electrode. Under this configuration, the total applied amplitude across the cell varies from −1.4*V* to −2.2*V* for the given pulse width of 200 *ns*. For each *V*_*stop*_ voltage, the cell is toggled to a different high resistance state (HRS). The final resistance state has been read by the means of a 120 *μs* pulse with amplitude *V*_*READ*_ = 0.1*V*. Figure 2(c) shows the mean value of each resistive state from R0 to R4.

**Figure 3:**
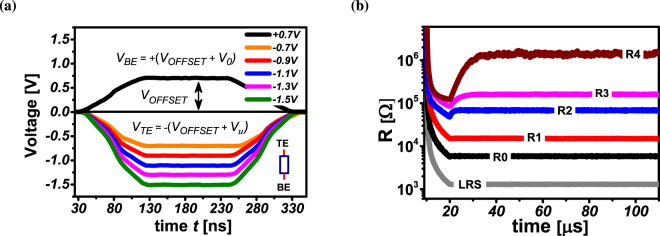
Primary logic operation. The logic operands *u* and *v* are applied to top (TE) and bottom (BE) electrode, respectively. Operand voltages *V*_*u*_ and *V*_*v*_ range from 0*V* to 0.8*V* in steps of 0.2*V*. Equal stepping of operand voltages is enabled using an OFFSET voltage *V*_*OFFSET*_ = 0.7*V*. (**a**) Keeping the *V*_*BE*_ = 0.7*V* constant, *V*_*TE*_ is varied from −0.7*V* to −1.5*V* in steps of −0.2*V* i.e. *V*_*v*_ = 0*V* and *V*_*u*_ = 0, 0.2, …, 0.8*V*. (**b**) The corresponding resistance levels *R*0, …, *R*4 states are programmed to the device. The actual resistive states are read by means of a 120 *μs* long voltage pulse with 0.1*V* (*V*_*READ*_) amplitude.

For Łukasiewicz logic family *L*_3_ (or three-valued Łukasiewicz logic family), three logic values are used. The three resistance states (R0, R1, R2) of the multi-level device correspond to logic values (0, 0.5, 1) respectively in the Łukasiewicz logic family *L*_3_. Additionally, the intermediate results in *L*_3_ can be (1.5 or 2) which require two additional logic values. The resistance states (R3, R4) represent the intermediate logic values (1.5, 2) respectively. Corresponding to the logic values *u* = {0, 0.5, 1, 1.5, 2}, the operand voltage *V*_*u*_ is {0, 0.2, 0.4, 0.6, 0.8}*V* respectively. The multi-valued operands say *u* and *v* can be applied as operand voltages to the top electrode (TE) and the bottom electrode (BE). Note that, *u* and *v* are always from the multi-valed logic set *L*_3_ {0, 0.5, 1}. A predefined OFFSET voltage *V*_*OFFSET*_ is used for each pulse to allow equidistant voltage stepping. The voltage applied to the TE is *V*_*TE*_ = −(*V*_*OFFSET*_ + *V*_*u*_). Depending on the operation being realized, the actual voltage applied to the BE is *V*_*BE*_ = *V*_*OFFSET*_ ± *V*_*v*_. The effective potential difference across the device is *V*_*eff*_ = *V*_*TE*_ − *V*_*BE*_. If *V*_*BE*_ = *V*_*OFFSET*_ + *V*_*v*_, the resulting resistance state of the device is *R*_*u*+*v*_. Otherwise if *V*_*BE*_ = *V*_*OFFSET*_ − *V*_*v*_, the resulting resistance state of the device is *R*_*u*−*v*_. Fig. 3 demonstrates the multi-level operation of the device.

### Proposed Łukasiewicz Logic Implementation

The developed implementation strategy for realization of the Łukasiewicz logic operates and stores the multi-valued results directly in the ReRAM devices. The computed results are available as the resistive states of the device and can be read out. Before any operation, the devices are initialized to LRS. The realization of the NEG¬ and IMP→ operator for *L*_3_ is explained below.

### NEG¬ operator

The negation operator works on a single operand. For computing ¬*u*, a constant voltage −(*V*_*OFFSET*_ + *V*_1_) is applied to the TE while the *V*_*OFFSET*_ − *V*_*u*_ is applied to the BE. The negated operand is stored in the ReRAM device as a corresponding resistive state.

### IMP→ operator

**Figure 4:**
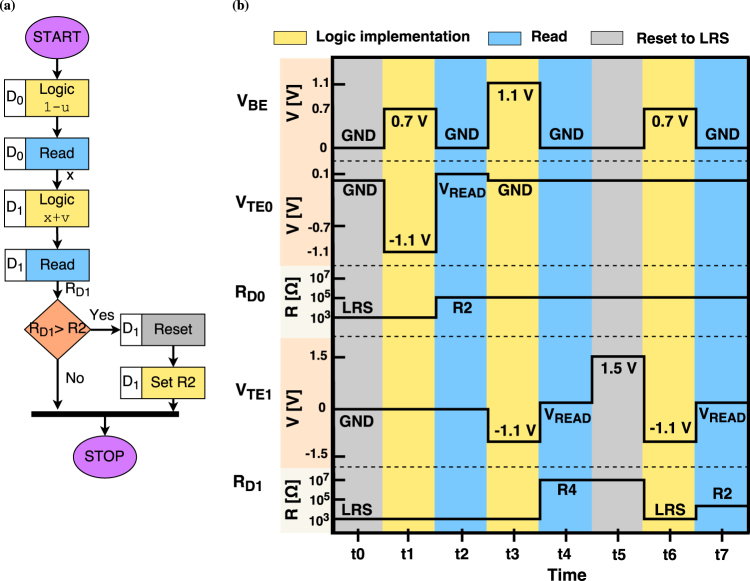
Implication implementation. (**a**) Flowchart for the implication (*u* → *v* = *min*(1, 1−*u* + *v*)) computation. First, the negation of operand *u*, i.e. 1 − *u*, is computed. Next, the logic operation is conducted to compute 1 − *u* + *v* in device *D*_1_. The device state *R* is read out from the *D*_1_. To compute *min*(1, 1 + *u* − *v*), we check whether *R* *>* *R*2 or not. If *R* > *R*2, then the device *D*_1_ is reset to the LRS and set to resistive state *R*2. (**b**) Implication computation *u* → *v* for *u* = 0 and *v* = 1. *V*_*BE*_ is the voltage sequence applied to the BE of the devices whereas *V*_*TE*0_ and *V*_*TE*1_ is the voltage applied to the TE of device *D*_0_ and *D*_1_ respectively. The transition of resistive state for device *D*_0_ and *D*_1_ is shown as *R*_*D*0_ and *R*_*D*1_ respectively. After the computation, the final resistive state of device *D*_1_ is the result of *u* → *v*, which in this case is equal to *R*2, corresponding to logic 1.

The implication operator works on two operands. The flowchart for performing the implication operation *u* → *v* is shown in Fig. 4(a) and the steps are described below.

**Step 1:** In the beginning, all the devices are initialized to the LRS.

**Step 2:** To compute ¬*u* in device *D*_0_, −(*V*_*OFFSET*_ + *V*_1_) is applied to the TE and *V*_*OFFSET*_ −*V*_*u*_ to the BE.

**Step 3:** The resistive state of the device *D*_0_ is read out by means of *V*_*READ*_ = 0.1*V*.

**Step 4:** In the second device *D*_1_, the negated sum of offset voltage *V*_*OFFSET*_ and the voltage corresponding to read out value *V*_¬*u*_ is applied to the TE i.e *V*_*TE*_ = −(*V*_*OFFSET*_ + *V*_¬*u*_) whereas at the BE, *V*_*BE*_ = *V*_*OFFSET*_ + *V*_*v*_ is applied.

**Step 5:** In the following step, resistive state *R* of the *D*_1_ is read out. If the resistive state *R* < *R*2, then the operation is complete. Otherwise, device *D*_1_ is toggled to the LRS and the set operation is used to change the device state to *R*2 i.e., *V*_*TE*_ = −1.1*V* and *V*_*BE*_ = 0.7*V* is applied.

Figure 4(b) demonstrates the computation of 0 → 1 using the proposed method in terms of applied operation voltages and corresponding states. The overall operation requires seven steps. Initially, the device *D*_0_ and *D*_1_ are in LRS state, shown as *R*_*D*0_ and *R*_*D*1_ respectively. In cycle *t*1, 1 − *u* is computed by applying 0.7*V* to the BE and 1.1 − *V* to the TE of device *D*_0_, shown as *V*_*BE*_ and *V*_*TE*0_ respectively. In cycle *t*2, the resistive state of *D*_0_ is read out, which is *R*2. In the next cycle, 1 − *u* + *v* is computed by applying 1.1*V* and −1.1 to the BE and TE of device *D*_1_. In cycle *t*4, the current resistive state(*R*_*D*1_) of device *D*_1_ is read out. Since *R*_*D*1_ = *R*4 > *R*2, device *D*_1_ is reset to the LRS and then set to *R*2 in cycles *t*5 and *t*6. The computation of 0 → 1 is complete. To verify the correctness of computation, we read out the state of the device *D*_2_ in the last cycle *t*7. The state *R*_*D*2_ is *R*2 (corresponding to logic 1) which is the correct result for 0 → 1.

### Proof-of-concept

**Figure 5:**
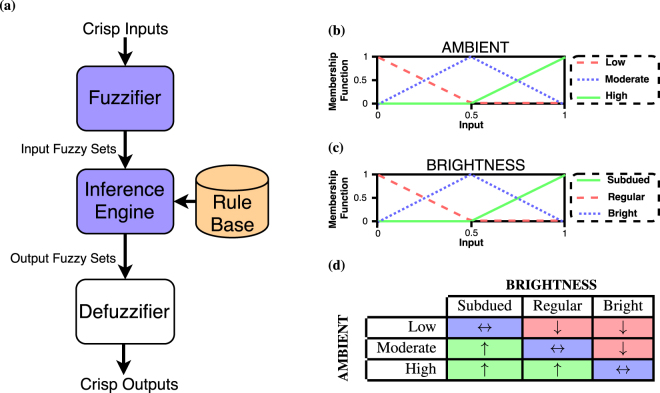
Fuzzy logic control. (**a**) Processing blocks used for fuzzy logic control are shown. The violet coloured blocks are implemented using the multi-state ReRAM devices. The rule base is stored as control/instruction steps. (**b**) Membership functions for variable *AMBIENT*. (**c**) Membership functions for variable *BRIGHTNESS*. (**d**) Rule table for fuzzy brightness controller.

We demonstrate the realization of a fuzzy logic controller as a representative application of Łukasiewicz logic. A conventional fuzzy controller has three major steps, as shown in Fig. 5(a).

1. Fuzzify input variables using fuzzy membership functions. If the inputs to the system are analog, analog-to-digital converters would be used to convert the inputs to the corresponding values in Łukasiewicz logic. Then, these multi-valued inputs are *fuzzified* using the membership functions.
2. Execute all the fuzzy inference rules from the rule database to determine the fuzzy output functions.
3. Defuzzify the fuzzy output functions to get *crisp* output value i.e a single multi-valued output value.

To illustrate the working of a fuzzy logic control, we consider a fuzzy logic controller for regulating screen brightness. The screen brightness has to be regulated based on the ambient light *AMBIENT* and screen brightness *BRIGHTNESS*. Each of the variables can be represented using three gradations.

- *AMBIENT* ∈ {Low (L), Moderate (M), High (H)}
- *BRIGHTNESS* ∈ {Subdued (S), Regular (R), Bright (B)}

We use the fuzzy membership functions shown in Fig. 5(b) and (c) to fuzzify the input variables *AMBIENT* and *BRIGHTNESS* respectively. The fuzzy membership functions can be expressed in terms of Łukasiewicz operators^32,33^. Therefore, we can realize the “Fuzzifier” block of a fuzzy control system (shown in Fig. 5(a)) using Łukasiewicz logic operations only. Let us consider the inverted notch membership function *f*_(*LOW*,*AMBIENT*)_ for the grade *LOW* of variable *AMBIENT*. *f*_(*LOW*,*AMBIENT*)_. It can be expressed as (¬*v* → *v*) → 0, where *v* is the input. Similarly, the flipped notch membership function *f*_(*SUBDUED*,*BRIGHTNESS*)_ for the grade *SUBDUED* of variable *BRIGHTNESS* can be written as (*v* → ¬*v*) → 0. These membership functions can be simplified and expressed in terms of *min*(*u*, *v*) and ¬*u* (1 − *u*) operation as shown below.

4 $${f}_{(LOW,AMBIENT)}=(\neg v\to v)\to 0=1-min\mathrm{(1,}\,2v)$$

5 $${f}_{(SUBDUED,BRIGHTNESS)}=(v\to \neg v)\to 0=1-min\mathrm{(1,}\,1-v+1-v)$$

**Figure 6:**
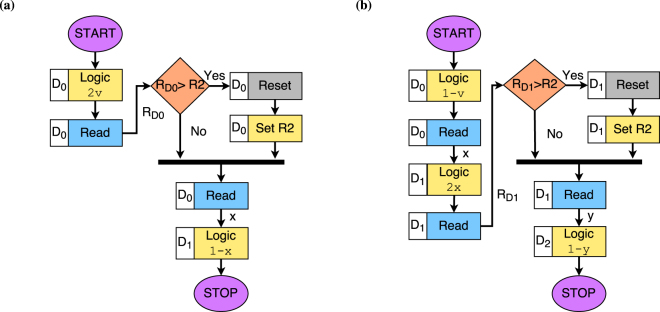
Realization of two fuzzy membership functions. (**a**) Steps to compute inverted notch[(¬*v* → *v*) → 0]. (**b**) Steps to compute flipped notch[(*v* → ¬*v*) → 0].

**Figure 7:**
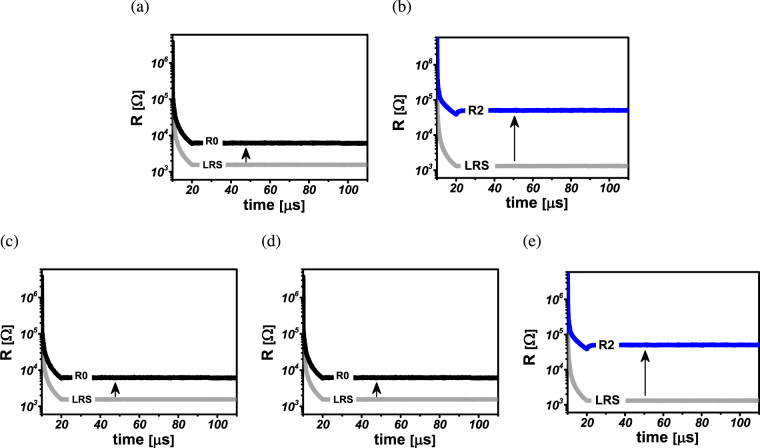
Membership function realization using multi-state ReRAM devices. (**a**,**b**) State transition of device *D*_0_ and *D*_1_ for realization of inverted notch membership function for *v* = 0. (**c**–**e**) State transition of devices *D*_0_, *D*_1_ and *D*_2_ for realization of flipped notch membership function for *v* = 1.

The detailed procedure to simplify the functions is provided in Supplementary Discussion S1. The series of steps required to realize the inverted notch and flipped notch membership function using multi-state ReRAM is presented in Fig. 6(a) and (b) respectively. We experimentally verified the correctness of computation. Figure 7 shows the experimental results when the input variable *AMBIENT* is 0 and also for value 1 for input *BRIGHTNESS*. In Supplementary Discussion S2, we have shown the experimental validation of membership function for other values of input *AMBIENT*.

**Figure 8:**
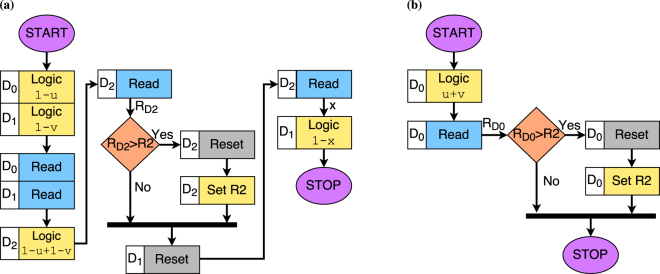
Fuzzy rule evaluation. (**a**) Computation of Łukasiewicz T-norm. (**b**) Computation of Łukasiewicz T-conorm.

**Figure 9:**
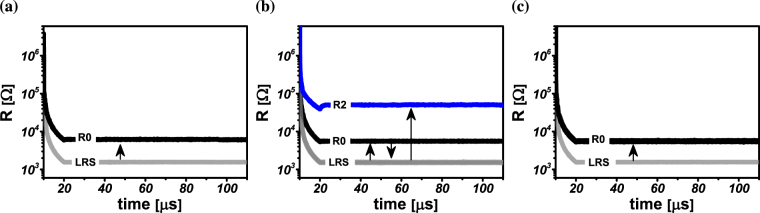
Łukasiewicz T-norm computation for *u* = 1 and *v* = 1. State transitions for (**a**) *D*_0_ (**b**) *D*_1_ and (**c**) *D*_2_ ReRAM device respectively.

The fuzzy inference engine determines the *ACTION* to be taken based on fuzzified inputs and be stated as a set of rules. The *ACTION* can be increase brightness (↑), decrease brightness (↓) or no action (↔). A rule for example can the following–if *AMBIENT* is Low and *BRIGHTNESS* is Subdued, then *ACTION* is no action (↔). Figure 5(d) presents multiple such control rules to determine the *ACTION* represented compactly as a rule table. To evaluate the output of a fuzzy rule, a suitable T-norm function is used. We use Łukasiewicz T-norm–*max*(0, *u* + *v* − 1) for evaluation of the fuzzy controller rules. The Łukasiewicz T-norm can be expressed in terms of *neg* and *min* functions as ¬*min*(1, (1 − *u*) + (1 − *v*)). Figure 8(a) show the sequence of steps to realize Łukasiewicz T-norm using the ReRAM devices. As a representative example, Fig. 9 shows the state transitions of the ReRAM devices during computation of T-norm for inputs *u* = 1 and *v* = 1.

Once all the rules have been evaluated, the outputs of these is combined using a suitable T-conorm. The T-conorm should be the dual of the T-norm used for rule evaluation. Therefore, we use Łukasiewicz T-conorm which is *min*(1, *a* + *b*), for combining the output of the rules. Figure 8(b) shows the steps for computation of the Łukasiewicz T-conorm using the multi-state memristive devices. T-conorm evaluation for inputs *u* = 1 and *v* = 0.5 is given in Supplementary Fig. S3. To obtain *crisp* output, the combined fuzzy output in the end has to be defuzzied. The defuzzifier block shown in Fig. 5(a) computes *crisp* output using an appropriate defuzzification method^34^. Note that the defuzzifier block has not been implemented in the presented prototype, which can be realized using conventional methods.

## Discussion

Knowledge-based system is capable to reason with judgmental, imprecise, and qualitative knowledge as well as with formal knowledge of established theories. The design of such systems is an important challenge in the realm of Artificial Intelligence (AI). The incompleteness and uncertainty associated with the knowledge-base in is handled through fuzzy logic. Fuzzy logic allows linguistic variable^35^ to be assigned inexact or partial truth values for modeling logical reasoning. In this work, we have shown the realization of fuzzy logic control in terms of three-valued Łukasiewicz logic operands. We have demonstrated the feasibility of implementation of three-valued Łukasiewicz logic *L*_3_ using the multi-states memristive devices.

Our demonstrated method can be scaled up for arbitrary *n*-valued Łukasiewicz logic *L*_3_, *n* ≥ 3, depending on the number of resistive states available. For the realization of *L*_*n*_, the memristive device should support at least 2*n* states. From the perspective of area, the implementation of a higher-valued logic system does not increase the area per device since it is dependent on the number of resistive states. However with increase in number of resistive states, the peripheral circuitry has to be more robust.todo.

Regarding the representation of numbers, it is well understood that for higher radix, the number of literals reduce in logarithmic order in comparison to lower-radix. For example, the efficiency of a *n*-valued representation of a truth-value *N* compared to its corresponding Boolean representation is equal to $\frac{{lo}{{g}}_{n}(N)+1}{{lo}{{g}}_{2}(N)+1}$. Implementation of a given fuzzy system in Boolean logic requires the treatment of every member with varied degree in a separate set and performing Boolean logic operations on those sets. Therefore, the computation steps do also increase in logarithmic proportion when using the Boolean logic in comparison to the fuzzy logic. Traditionally, Mamdani-type fuzzy systems use *min* and *max* functions for evaluation of fuzzy rules and combining the output of the rules^11^. *min* and *max* functions can be expressed as in terms of Łukasiewicz logic operators:

6 $$min(u,v)=(u\to v)\to v$$

7 $$max(u,v)=\neg (min(\neg u,\neg v))$$

Therefore, it is possible to use the multi-valued Łukasiewicz logic for realization of Mamdani type fuzzy systems as well. In general, Łukasiewicz logic is capable of dealing with a wide range of approximate reasoning paradigms, since it can express evaluation function of multi-valued logic classes described in terms of +, *−*, *min* and *max* ^35–37^.

In past, Łukasiewicz logic arrays has been demonstrated for realization of fuzzy inference engines and expert systems^38–40^ with CMOS-based circuitry. However, such realizations did not have any multi-valued storage devices for storing the intermediate results thereby requiring costly conversions to-and-from binary representation. In this work, we have experimentally realized a working fuzzy system by using Łukasiewicz logic, that does not use any intermediate binary/Boolean representation. Although implementation of fuzzy logic gates have been reported in the DNA computing paradigm^41^, this is the first experimentally reported work on multi-valued logic operators as well as a demonstrative application of that in fuzzy inference engine using memristive devices. Recently, an implementation of Boolean minimum and maximum gate has been demonstrated using memristive devices^42^ with their application restricted to the implementation of sorting networks.

Here, we have successfully demonstrated Łukasiewicz logic operation on 2 *μm* × 2 *μm* ReRAM devices. However, these devices are fully compatible to 4*F* ^2^ configuration in crossbar array in conjunction with a selector device and can be scaled down to 5 *nm* ^43,44^. The integration of the selector device would prevent the problem of sneak paths in the crossbar array. Ultra-dense large-scale multi-state ReRAM crossbars can be controlled by peripheral control circuitry, as shown in Supplementary Fig. S4. The approach of implementing Łukasiewicz logic operation within the resistive memory device using the available multi-level states is a highly attractive option for future hybrid CMOS/ReRAM chips for enhancing its present functionality. Each multi-valued operation requires a constant number of steps, 1 step for negation and 7 steps for implication (depicted in Fig. 4), to be realized, irrespective of the value of *n* in an *n*-valued logic system. For Boolean realization of the implication and negation operators, the number of steps would increase with the value of *n* ^29,45,46^. Furthermore, parallel operations across multiple devices that share the same wordline, can be enabled by carefully packing operations that have the same input, similar to the strategy proposed by Bhattacharjee *et al*.^47^. In contrast, to leverage such parallelism, the Boolean circuits corresponding to the implication and negation operations need to be replicated. Multi-level ReRAM devices reduces the complexity of state representation and thus, brings fundamental benefits across arithmetic and logical primitives. This capability has far-reaching implications in modern Internet-of-Things (IoT) systems, which promotes local computing due to the bandwidth scarcity. By having multi-valued and fuzzy logic primitives at the device level, efficient processing-in-memory can be undertaken for application domains like public key cryptography, error correcting codes, industrial control and security. Note that, the energy-efficiency can be further boosted by having short-pulse (sub-*ns*) operations.

## Conclusion

Fuzzy set allows its elements with certain degrees of membership, in contrast to a crisp set. Operators on the fuzzy set can realistically model real-life applications in, for example, industrial control, linguistics, decision variables and bio-informatics, and therefore, have grown in usage over last half century. The logic operations on the fuzzy set is performed through fuzzy inference system. In this manuscript, we demonstrated a practical fuzzy inference system, by realizing the fuzzy logic operations using the multi-state *TaO*_*x*_ devices. Further, each fuzzy operation is mapped to a series of the multi-valued logic primitives, namely, Łukasiewicz logic. We showed a practical fuzzy inference system through a limited number of logical steps and 1 × 3 memristive crossbar array. The multi-state *TaO*_*x*_ devices enable computation entirely using multi-valued elements for the operations, without need for any intermediate representations. Therefore, these devices provide a natural platform to undertake multi-valued logic and thus, fuzzy inference operations. We believe that these results can greatly benefit scientific community and provide a direction to move forward in the field of fuzzy logic.

## Methods

### Device Fabrication

Cross-point based *Ta*_2_ *O*_5_ ReRAM was fabricated on thermally grown *SiO*_2_ samples. In our design, each device shares a common bottom electrode (BE). The BE was patterned in 30 *nm*-thick platinum (*Pt*) layers, grown by the sputtering process. After patterning the BE, switching layer of 7 *nm*-thick *TaO*_*x*_, 13 *nm*-thick tungsten (*W*), and 25 *nm*-thick platinum (*Pt*) were sequentially deposited by the sputtering process. The *TaO*_*x*_ layer was grown with reactive sputtering process with 76.6% Argon and 23.3% Oxygen at partial pressure of 2.3 × 10^−2^ *mbar*. The *W* ohmic electrode, and the *Pt* were grown with DC sputtering method. For the top electrode (TE) patterning, photo-lithography and reactive ion etching steps were performed. These steps lead to the *Pt*/*W*/*TaO*_*x*_/*Pt* memristive device stack. Figure 1 shows the scanning electron microscopy (SEM) of 1 × 3 crossbar array with 2 *μm* × 2 *μm* size cell with cross-sectional tunneling electron microscopy (TEM) image and its corresponding schematic diagram. More experimental details can be found in reference^48^.

### Measurement Set-up

The pristine state of the memristive devices was highly resistive (*G*Ω) and therefore an electroforming process was required. This process was carried out by applying a positive DC voltage on the TE for a given current compliance, while keeping the BE grounded. This turned the devices into low resistance state (LRS). Now, the memristive devices were sequentially switched to high resistance state (HRS) by the ‘reset process’ and low resistance state (LRS) by the ‘set process’. In this experiment, the ‘set’ process is performed with DC voltage while the reset operations were performed with 200 *ns* pulse width and 120 *μs* long pulse width at 0.1*V* was used to read the respective resistance states. More measurement details can be traced in reference^48^.

### Data availability

No datasets were generated or analysed during the current study.

## Electronic supplementary material

Supplementary information
