# Supplementary material for: Multi-valued and Fuzzy Logic Realization using TaOx Memristive Devices
Source: Sci Rep. 2018 Jan 8;8:8. doi: 10.1038/s41598-017-18329-3 (PMC5758575; doi:10.1038/s41598-017-18329-3)
Supplement: Supplementary file 1 — Supplementary information [file 41598_2017_18329_MOESM1_ESM.pdf]

SUPPLEMENTARY INFORMATION
Multi-valued and Fuzzy Logic Realization using TaO_x Memristive Devices

Debjyoti Bhattacharjee[1], Wonjoo Kim[2], Anupam Chattopadhyay[1,3], Rainer Waser[2,4], and Vikas Rana[2,*]

[1]School of Computer Science and Engineering, Nanyang Technological University, Singapore
[2]Peter Grünberg Institut 7, Forschungszentrum Jülich GmbH, 52425 Jülich, Germany
[3]School of Physical and Mathematical Sciences, Nanyang Technological University, Singapore
[4]Institut für Werkstoffe der Elektrotechnik II, RWTH Aachen University, 52074 Aachen, Germany
[*]Corresponding author (v.rana@fz-juelich.de)

Supplementary S1: Simplification of some multi-valued functions is presented below.

- Inverted notch function.

$$
\begin{aligned}
(\neg v \rightarrow v) \rightarrow 0 &= (min(1, 1-(1-v)+v)) \rightarrow 0 && \text{(SE 1)}\\
&= min(1, 2v) \rightarrow 0 && \text{(SE 2)}\\
&= x \rightarrow 0, \quad \text{where } \ x = min(1, 2v) && \text{(SE 3)}\\
&= min(1, 1-x+0) && \text{(SE 4)}\\
&= min(1, 1-x) && \text{(SE 5)}\\
&= 1-x && \text{(SE 6)}
\end{aligned}
$$

- Flipped notch function.

$$
\begin{aligned}
(v \rightarrow \neg v) \rightarrow 0 &= (min(1, 1-v+1-v)) \rightarrow 0 && \text{(SE 7)}\\
&= min(1, x) \rightarrow 0, \quad \text{where } \ x = 1+v+1-v && \text{(SE 8)}\\
&= min(1, 1-min(1-x)+0) && \text{(SE 9)}\\
&= min(1, 1-min(1-x)) && \text{(SE 10)}\\
&= 1-min(1-x) && \text{(SE 11)}
\end{aligned}
$$

- Łukasiewicz T-norm.

$$
\begin{aligned}
max(0, u+v-1) &= \neg\neg max(0, u+v-1) && \text{(SE 12)}\\
&= \neg min(1, 1-(u+v-1)) && \text{(SE 13)}\\
&= \neg min(1, (1-u)+(1-v)) && \text{(SE 14)}
\end{aligned}
$$

Supplementary S2: Inverted notch membership function realization for inputs $v = 1$ and $v = 0.5$. For input $v = 1$, the function can be evaluated theoretically as follows.

$$v = 1 \quad \text{(SE 15)}$$
$$(\neg v \rightarrow v) \rightarrow 0 = min(1, 1 - min(1, 2v)) \quad \text{(SE 16)}$$
$$= min(1, 1 - 1) \quad \text{(SE 17)}$$
$$= 0 \quad \text{(SE 18)}$$

The set of steps required to do the computation for $v = 1$ using the multi-state memristive devices is shown below. The corresponding state transitions for devices are shown in Supplementary Fig. S1. Similarly during computation of inverted knotch function with input $v = 0.5$, the corresponding state transitions are shown in Supplementary Fig. S2.

Cycle	Device	TE	BE	Description
$t1$	D_0	$-1.10V$	$1.10V$	Compute $2v$
$t2$	D_0	$0.1V$	$0V$	Read Device D_0.
$t3$	D_0	$1.5V$	$0V$	Reset Device D_0 to the LRS.
$t4$	D_0	$-1.1V$	$0.7V$	Set Device D_0 to R_1.
$t5$	D_0	$0.1V$	$0V$	Read Device D_0.
$t6$	D_1	$-0.7V$	$0.7V$	$1 - x$.
$t7$	D_1	$0.1V$	$0V$	Read Device D_1.

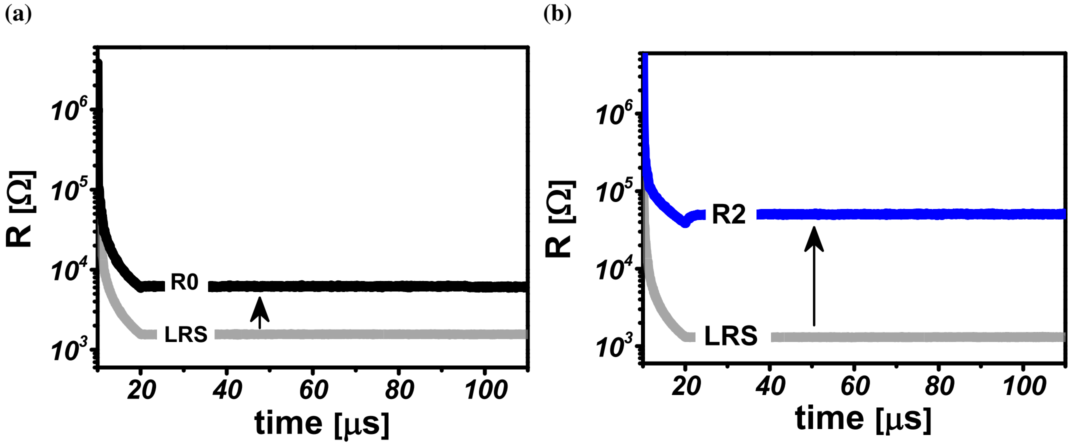

Figure S1. State transitions for device (a) D_0 and (b) D_1 for computation of inverted notch membership function for $v = 1$.

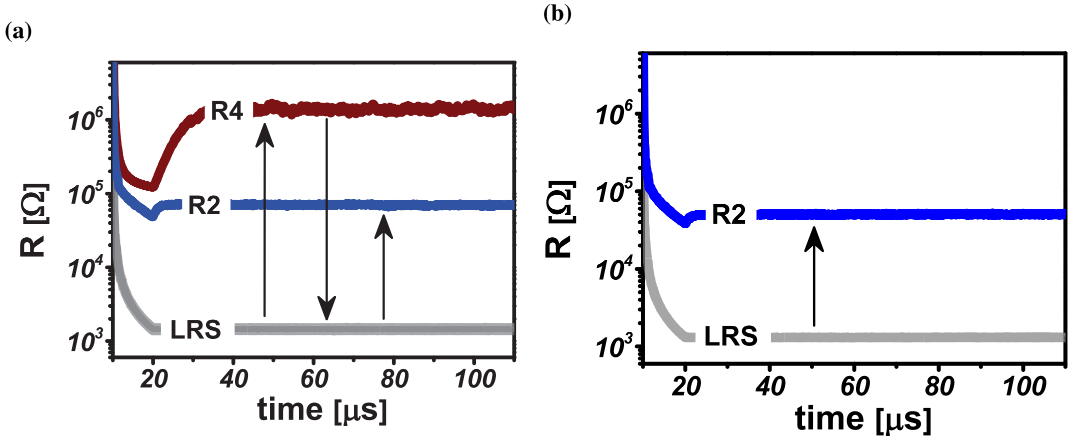

Figure S2. State transitions for device (a) D_0 and (b) D_1 for computation of inverted notch membership function for $v = 0.5$.

Supplementary S3:

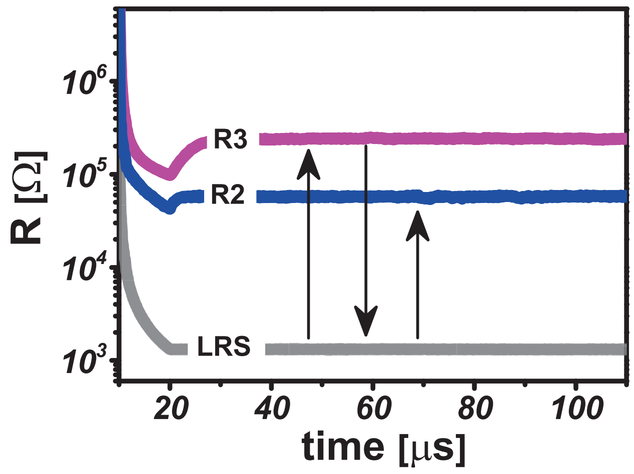

Figure S3. State transition in device D_0 for computation of Łukasiewicz T-conorm for inputs $u = 1$ and $v = 0.5$.

Supplementary S4:

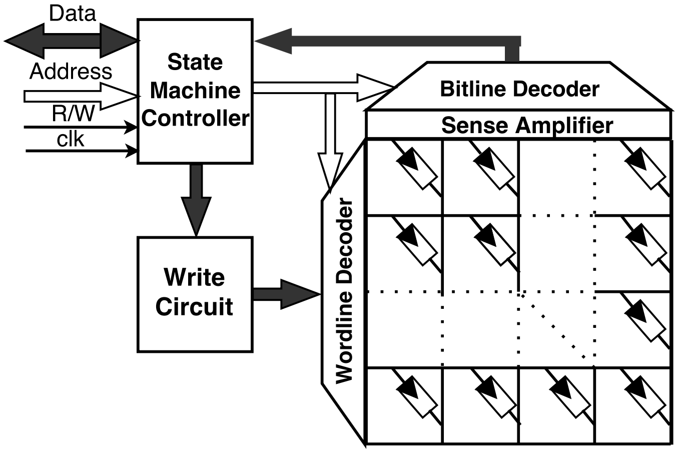

Figure S4. A schematic of controller circuit to enable multi-valued operations using multi-state ReRAM arrays.
